# Age, sex and storage time influence hair cortisol levels in a wild mammal population

**DOI:** 10.1371/journal.pone.0221124

**Published:** 2019-08-09

**Authors:** Alexandre Azevedo, Liam Bailey, Victor Bandeira, Martin Dehnhard, Carlos Fonseca, Liliana de Sousa, Katarina Jewgenow

**Affiliations:** 1 Department of Reproduction Biology, Leibniz Institute for Zoo and Wildlife Research, Berlin, Germany; 2 Instituto de Ciências Biomédicas Abel Salazar, Porto, Portugal; 3 Department of Biology & CESAM, University of Aveiro, Aveiro, Portugal; Centre for Cellular and Molecular Biology, INDIA

## Abstract

The measurement of hair cortisol is increasingly used to understand the effect of natural and anthropogenic stressors on wild animals, but it is potentially confounded by individual, seasonal and sex-dependant variations in baseline cortisol secretion. This study validated an enzyme-linked immunoassay for hair cortisol measurement and characterized its baseline variation in a wild population of Egyptian mongoose. The analysis encompassed individuals of both sexes and all ages, across a range of geographic, environmental and seasonal conditions that the species experiences in Portugal allowing us to account for spatial, temporal and biological factors that contribute to hair cortisol variation. Our results showed that age, sex and storage time had an effect on hair cortisol, but season did not. Hair cortisol was higher in early stage juveniles compared to other age cohorts, in males when compared to females, and decreased with longer storage time. By identifying the factors that influence baseline hair cortisol in this wild population, we establish the basis for its application as an indicator of the effect of natural and anthropogenic stressors.

## Introduction

In the Anthropocene, wild animal populations are faced with a broad range of environmental stressors from a mix of both anthropogenic and natural sources. Although consequences such as species declines and ecosystem imbalances are often measurable, causal mechanisms underlying conservation problems are more difficult to establish and require an understanding of the physiological responses of animals to environmental change [1]. This has led to the emergence of the field of conservation physiology and to a growing interest in understanding how environmental change affects the physiology of wild animal populations, with important implications for wildlife management and conservation. One way to study the effects of environmental stressors is through the measurement of substances that mediate the physiological stress response. In mammals, cortisol is a key mediator of the hypothalamic-pituitary-adrenal stress response. While acute changes in its circulating levels are necessary to maintain homeostasis in face of a dynamic environment, chronic elevations may impact immunity, behaviour and reproduction [2–4]. In controlled settings, cortisol is often measured in blood plasma and serum, but this only provides a snapshot of current cortisol levels, which can be influenced by the stress of the sampling procedure [4,5]. In addition strong ultradian rhythms with periods around 120 min or less may result in extreme fluctuations within individuals over time [6,7]. Consequently, non-invasive approaches have been developed using alternative sample matrices such as urine, faeces or saliva to measure glucocorticoid metabolites that mirror adrenocortical (stress) status of the preceding hours or days [4,5]. More recently, the measurement of cortisol in hair has been used to provide an integrated value of circulating cortisol over a period of several weeks [8–14]. The exact sources of cortisol in found hair are not fully understood and include both incorporation of circulating cortisol from blood vessels and tissues surrounding follicular cells in growing hair [15] and production by a local HPA-axis analogue [16–18]. Nevertheless, evidence that hair cortisol reflects changes in circulating levels in cases of hypoadrenocorticism [19], hyperadrenocorticism [20], consecutive ACTH injections [11,14] and major life stressors [10,21] indicates that circulating cortisol levels are reflected in hair. Finally, this approach offers great potential as an indicator of chronic stress because it is unaffected by sampling procedures, ultradian rhythms or globulin-bound cortisol [22].

Before hair cortisol can be broadly used as an indicator of stress, it is first necessary to investigate how much of observed hair cortisol variations can be attributed to stress and disturbance in relation to baseline and individual variation. Each individual’s baseline adrenocortical activity and HPA-axis reactivity is influenced by genetic inheritance [23,24], maternal and epigenetic effects [25–27], biotic environmental factors like infection or predation risk [28,29] and abiotic factors like weather and climate [30,31]. Additionally, glucocorticoid levels may vary with sex [32], developmental stage [33,34] and age [11,35]. Although often not evident in studies using captive animals, seasonal variations in glucocorticoids levels have been demonstrated in wild species [36,37]. Highest levels of glucocorticoids are mostly related with the breeding season in birds and reptiles but in mammals this trend is less clear, with some species showing higher glucocorticoid levels during breeding season and others showing post-breeding increases [37,38]. In addition, several methodological issues need to be considered, since storage time, storage conditions, anatomical location [9,12,14] and type [12] and colour of the hair sample [11] have been shown to influence the amount of cortisol retrieved. Failure to account for these sources of variation in glucocorticoid levels may confound their interpretation and lead to a misestimation of the effects of disturbance.

The Egyptian mongoose (*Herpestes ichneumon*) is a medium-sized carnivore that colonized the Iberian Peninsula in the Pleistocene [39] and has experienced a recent expansion in its distribution due to several biotic and abiotic factors [40–42]. The species has a polygynic mating system, with different levels of investment by males and females in reproduction [43,44], leading to physiological and behavioural differences between sexes that might also be reflected in hair cortisol levels, especially during the breeding season. The widespread distribution and ongoing expansion of *H*. *ichneumon*, covering almost all types of habitat in Portugal [42], coupled with the availability of captured specimens [45,46], makes it a useful model for the study of hair cortisol as an indicator of stress.

In this study we determined hair cortisol in samples from wild caught Egyptian mongoose specimens with known sex, age, location and date of capture. We hypothesize that age, sex, and season will have an effect on hair cortisol variation, with effects of age and season likely to vary between sexes. We also considered how female reproductive state (non-breeding, lactating, pregnant) might affect hair cortisol measurements, with higher hair cortisol levels expected in breeding individuals. Through this work we aim to describe baseline variation in hair cortisol levels, which will contribute to a better understanding of the potential for hair cortisol to be used as an indicator of stress in wild animal populations. In addition HPLC analyses was carried out to investigate the profile of glucocorticoids that cross-react with our cortisol antibody as data from Keckeis et al. [47] demonstrated large amounts of immunoreactivity not coinciding with cortisol suggesting a local production of glucocorticoids in hair follicles.

## Methods

### Sample collection

We obtained hair samples from carcasses of wild Egyptian mongoose that were obtained from hunting activities in seven provinces of mainland Portugal between January 2008 and December 2014, in compliance with legal requirements and with permits from the competent authorities in Portugal, the ICNF–Instituto da Conservação da Natureza e das Florestas [45]. Following death, specimens had remained frozen at -20°C until the date of sample collection. After thawing, hair was clipped with scissors as close to the skin as possible from a standard area between the shoulders to account for variation in cortisol levels due to anatomical location. Samples were stored in paper envelopes in a dark dry location until the date of extraction. Storage time was defined as the total number of days between the date of capture of the mongoose and the date of cortisol extraction from hair.

Age was obtained based on dental development, with each mongoose classified as an adult (over one year of age), sub-adult (between nine and twelve months), type II juvenile (between five-and-a-half and nine months) and type I juvenile (between two-and-a-half and five-and-a-half months of age), following methods of Bandeira et al. [45].

Each specimen was assigned as male or female based on the presence of testicles or ovaries, and the reproductive state of females was noted as pregnant (foetuses identified in the uterus), lactating (milk present in mammary glands) or non-breeding.

Sample season was assigned based on the date of capture. In confirmation with other ongoing studies, animals captured from October to December were included in the autumn class, from January to March in the winter class, from April to June in the spring class and from July to September in the summer class.

A total of 294 Egyptian mongoose hair samples were collected for this study. Age cohort could not be determined for 50 specimens, which were excluded from the statistical analysis. Of the 244 mongooses with known age class, 114 were males and 130 were females (12 of which were pregnant and 7 lactating). In terms of age cohorts, 147 were adults, 27 were sub adults, 40 were type II juveniles, and 30 were type I juveniles. The number of specimens in each of the 7 provinces varied between 2 (Estremadura) and 134 (Baixo Alentejo) and sample storage time varied from 863 to 2,266 days.

### Sample preparation

All chemical reagents were purchased from Sigma–Aldrich (Taufkirchen, Germany) unless stated otherwise and were of the highest purity available.

Approximately 20 mg of full-length guard hairs were manually separated from undercoat hairs and placed in Eppendorf tubes. Guard hairs and undercoat are clearly distinguishable in Egyptian mongoose hair samples. In order to avoid variation due to the incorporation of different proportions of each hair type, we decided to use only one type of hair. Guard hairs were chosen because they are more likely to be retrieved from hair traps in future applications than undercoat, and allow comparison with similar studies in other species [e.g. 12].

To remove surface contamination, 2 mL of 90% methanol was added to the hair sample and vortexed for 5–10 seconds. Following settling, methanol was discarded and the wash step was repeated. After the two washes, samples were dried for one hour at 70°C. Next, 10 mg (8.29–12.12 mg) of washed hair were removed, ceramic beads (six 2.8 mm beads and 600 ± 10 mg of 1.4 mm beads) were added, and hairs were ground to a fine powder in a Precellys24 tissue homogenizer (Bertin Technologies, France). For cortisol extraction, 400 μL of 90% methanol were added to 10 mg of hair powder in separate tubes and shaken at room temperature for 30 minutes using a universal shaker (SM-30, Edmund Buhler GmbH, Hechingen, Germany). Samples were centrifuged (3 min, 1000G), the supernatant was collected and transferred to a new tube, diluted 1:2 with water and frozen until the day of cortisol measurement.

### High-performance liquid chromatography (HPLC)

To confirm cortisol as the major hair glucocorticoid in the Mongoose, hairs extracts were used for HPLC analysis. To obtain an appropriate cortisol concentration for HPLC analysis 150 μL of 294 hair extracts (in 40% methanol) corresponding to 550 mg hair were pooled and purified on a C18 column (ecf, Chromabond, Macherey–Nagel, Düren, Germany). For this purpose the pooled extract was diluted to 10% methanol with water. The C18 column was equilibrated with 2 mL 100% methanol followed by 2 mL of 20 mM Tris buffer (pH 8.5) containing 10% methanol. After applying the pooled hair extract on the C18 column it was washed twice with 2 mL of 20 mM Tris buffer (pH 8.5) containing 10% methanol. The purified sample was eluted with 3 mL of 100% methanol, evaporated in a sample concentrator (Dri Block DB3, Techne, Staffordshire, UK) under a constant nitrogen flow and finally resuspended in 120 μL of 100% methanol and 180 μL of water resulting in 300 μL purified extract in 40% methanol.

HPLC analysis was performed as described before [48]. In brief, 150 μL of the purified extract were separated on a reversed-phase Ultrasep ES ⁄RP– 18 ⁄ 6 μm HPLC column (4x250 mm; Sepserv, Berlin, Germany). Glucocorticoids were separated using a methanol + water mixture with the following gradient: 60% methanol over 5 min, 60–90% methanol over 10 min, 90% methanol for another 10 min. The flow rate was 1 mL/min. Fractions of 0.33 mL were collected at 20 sec intervals over a period of 25 min and diluted with one volume of water. All fractions were lyophilized and resuspended in 200μL 40% methanol. The elution positions of authentic cortisol, hydrocortisone, corticosterone, cortisone, testosterone, dihydrotestosterone, 11β-hydroxyetiocholanolone and progesterone had been previously determined in separate HPLC runs.

### Cortisol measurement

Cortisol was quantified by an enzyme immunoassay (EIA) using a polyclonal antibody (rabbit) against cortisol-3-CMO-BSA and cortisol-3-CMO-peroxidase as label. The antibody cross-reactivities to different steroids were as follows: 4-pregnen-11α,17,21-triol-3,20-dione (cortisol), 100%; 5α-pregnan-3β,11β,17,21-tetrol-20-one (3β,5α-tetrahydrocortisol), 8.4%; 4-pregnen-11β,21-diol-3,20-dione (corticosterone), 6.3%; 5α-pregnan-11β,17,21-triol-3,20-dione (5α-dihydrocortisol), 3.2%; and <0.1% for 4-pregnen-21-ol-3,20-dione (desoxycorticosterone), 5β-pregnan-11β,17,21-triol-3,20-dione (5β-dihydrocortisol), 5α-pregnan-3α,11β,17,21-tetrol-20-one (5α-tetrahydrocortisol), 5β-pregnan-3α,11β,17,21-tetrol-20-one (5β-tetrahydrocortisol), 4-pregnen-3,20-dione (progesterone), 5α-pregnan-3,20-dione, 5α-pregnan-3β-ol-20-one, dexamethasone, estradiol, and testosterone [49]. Duplicates of 10 μl hair extract or cortisol standards prepared in 40% methanol ranging from 0.2 to 100 pg/20 μl were then simultaneously pipetted into respective wells along with 100 μl cortisol-HRP conjugate in assay buffer (50 mM Na_2_HPO_4_/Na_2_HPO_4_, 0.15 M NaCl, 0.1% BSA, pH 7.4) with the aid of a diluter dispenser. Then, 100 μl of cortisol-specific antibody in a final dilution of 1:400.000 were added. After overnight incubation at 4°C, the assay was terminated following our standard protocol, described in Finkenwirth et al. [50]. Assay validation was performed by demonstrating parallelism of serially diluted hair extracts to the cortisol standard curve.

### Statistical methods

#### Effects of age, sex, season, and storage time

Statistical analyses were conducted in R (v3.5.1) using linear mixed effects models with a Gaussian error distribution from the package lme4 [51]. We considered the effect of mongoose age and sex, the season in which samples were collected and the storage time (days) on hair cortisol concentration (pg/mg). We also considered the possibility for interactions between sex and both season and age. Our model included a random intercept term for the province in which samples were collected (7 levels), and both the year and month in which samples were collected (12 months within each of 5 years) to account for potential spatial and temporal variation in hair cortisol concentration. Intercepts of random effects were assumed to follow a Gaussian distribution.

Variance inflation factors were used to test for multi-collinearity between variables. No evidence of multi-collinearity was detected using a variance inflation factor cut-off of 3. We identified two outlier values that were more than four times larger than median hair cortisol and almost twice as high as the next highest measurement (Cook’s distance of 0.38 and 0.63). Four-fold increases in hair cortisol have previously been observed in response to repeated ACTH-challenge in dairy cattle and eastern chipmunks (11,13), and so these values may be biologically plausible in situations of chronic and severe stress. We conducted all analyses with and without these outliers included. Results with outliers removed are presented in the main text while results with and without outliers included are provided in tables.

#### Effect of female reproductive state

Female reproductive state (non-breeding, pregnant, lactating) may also be an important factor affecting hair cortisol concentration. Therefore, we also fitted a general linear mixed effects model using female data only (N = 130). We considered the effect of female age and reproductive state, the season in which samples were collected and the storage time of samples (days) on hair cortisol concentration (pg/mg). The random effects structure of the model was the same as above.

#### Significance testing

Variable significance (α = 0.05) was determined using parametric bootstrapped likelihood ratio tests with 5,000 iterations using the package pbkrtest [52]. The parametric bootstrapping approach conducts multiple simulations of a likelihood ratio test and returns the fraction of simulated likelihood ratio test values that are larger or equal to the observed likelihood ratio test statistic taken from the true data (i.e. a significant result is one where the observed likelihood ratio test value is lower than 5% of simulated likelihood ratio test values). A model with each fixed effect parameter removed was compared to the global model containing all variables. Models were fitted using maximum likelihood during model comparison.

For each model we also calculated the repeatability of our random intercept terms (i.e. the amount of variance in hair cortisol concentration not explained by model fixed effects that can be attributed to consistent differences between provinces/years/months) with confidence intervals determined using parametric bootstrapping in the package rptR [53] with 5,000 iterations. We determined both the marginal R^2^ (variance explained by fixed effects) and conditional R^2^ (variance explained by fixed and random effects) following the procedure of Nakagawa and Schielzeth [54].

## Results

### HPLC analyses of hair glucocorticoids

**Fig 1:**
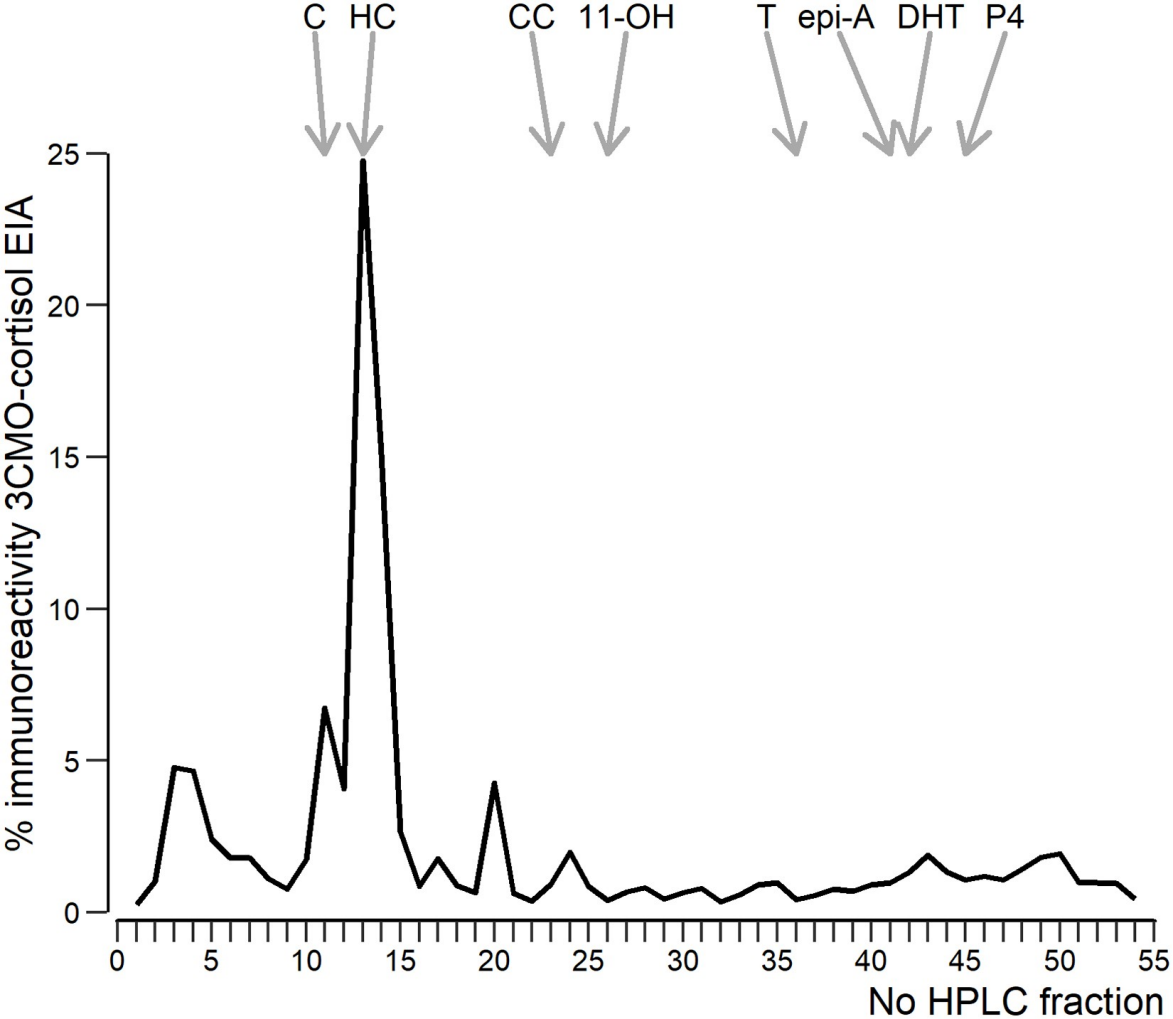
High performance liquid chromatography (reversed phase) separations of immunoreactive cortisol metabolites in pooled hair samples from Egyptian mongoose. The obtained fractions were analysed with a cortisol‐3‐CMO EIA. The elution positions of reference standards are indicated by arrows: 11: C (cortisone); 13/14: HC (cortisol); 23: CC (corticosterone); 26: 11‐OH (11‐hydroxyetiocholanolone); 36/37: T (testosterone); 41: epi‐A (epi‐androsterone); 42: DHT (dihydrotestosterone); 45: P4 (progesterone).

Analysis of HPLC fractions from the hair extract pooled from 294 individuals confirms cortisol as the major glucocorticoid in hairs from the mongoose (Fig 1) eluting in fractions 13 and 14. That coincides with the position of authentic cortisol corresponding to 40% of total immunoreactivity. Furthermore, three minor immunoreactive glucocorticoid metabolites peaks became visible in fractions 3–5, 11, and 20. From those, the immunoreactive compound in fraction 11 coincides with the position of authentic cortisone whereas the two remaining components co-elute with none of the steroid standards applied on column in previous runs. The most polar compound eluting in fractions 3–5 might indicate a cross-reacting glucocorticoid conjugate.

### EIA of hair cortisol

The mean cortisol concentration detected in Egyptian mongoose guard hair was 19.99 ± 8.52 pg/mg (ranging from 8.07 pg/mg to 114.18 pg/mg). The inter-assay coefficients of variation were 10.78% for extracts containing low and 15.95% for extracts containing high concentrations of cortisol. The intra-assay coefficients were 6.72% (n = 16) for extracts containing low and 5.37% (n = 16) for extracts containing high concentrations of cortisol. The sensitivity of the assay was 0.40 pg/well. Hair cortisol concentrations of two animals which were obtained from different provinces and years were statistically compatible with outliers, but within biologically plausible ranges according to ACTH stimulation tests done in other species. One was a sub-adult male collected in spring and the other was a non-breeding adult female collected in winter. There was no aspect of their data that allowed us to relate them and to explain the high level of cortisol.

### Effects of age, sex, season, and storage time

**Fig 2:**
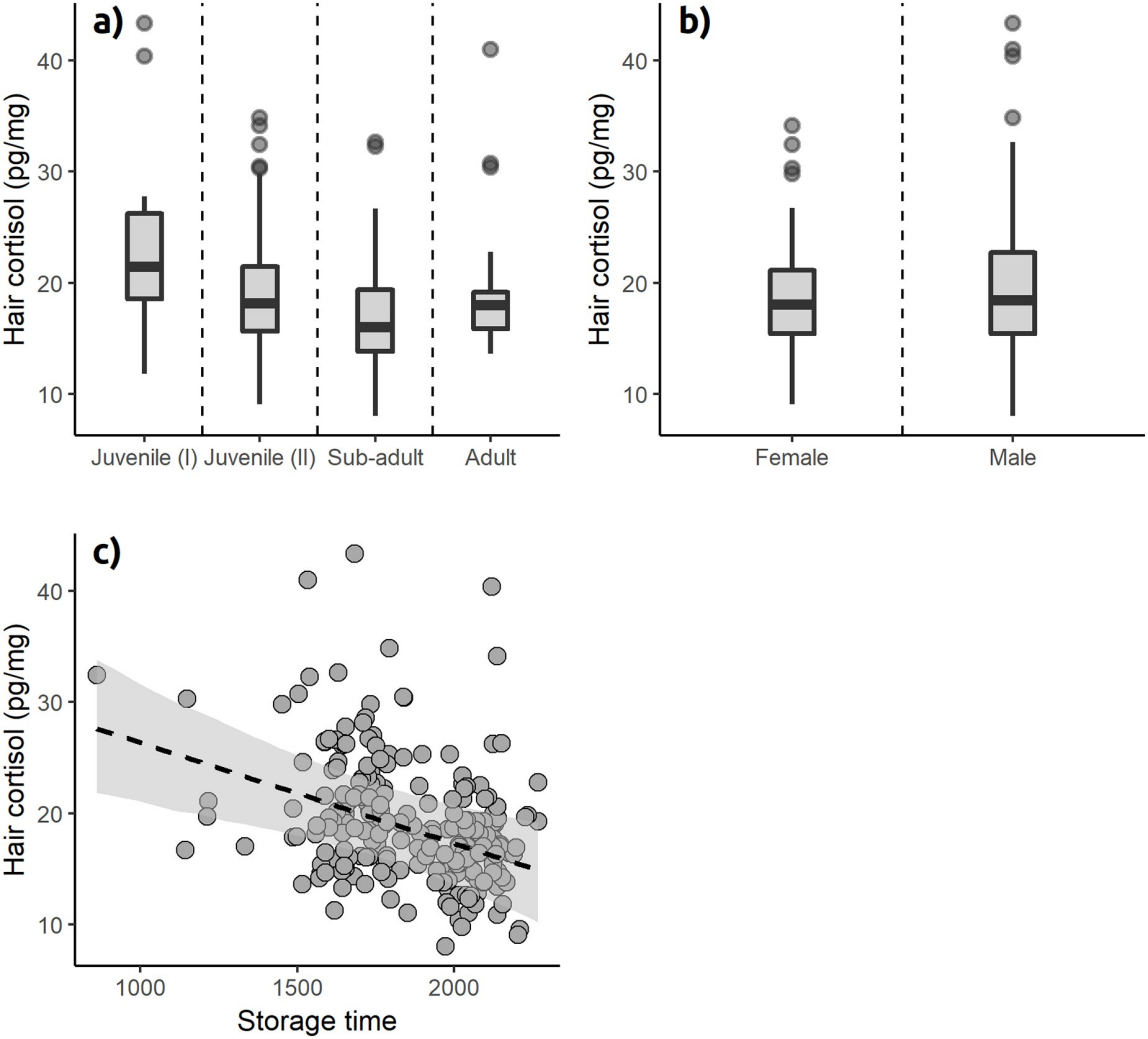
Variation in hair cortisol (pg/mg) in the Egyptian mongoose with a) age, b) sex, and c) storage time of hair samples (days). Hair cortisol concentration was higher in first stage juveniles than other age groups. Hair samples from males had higher cortisol concentration than females. Cortisol concentration was lower in hair samples stored for more days.

**Fig 3:**
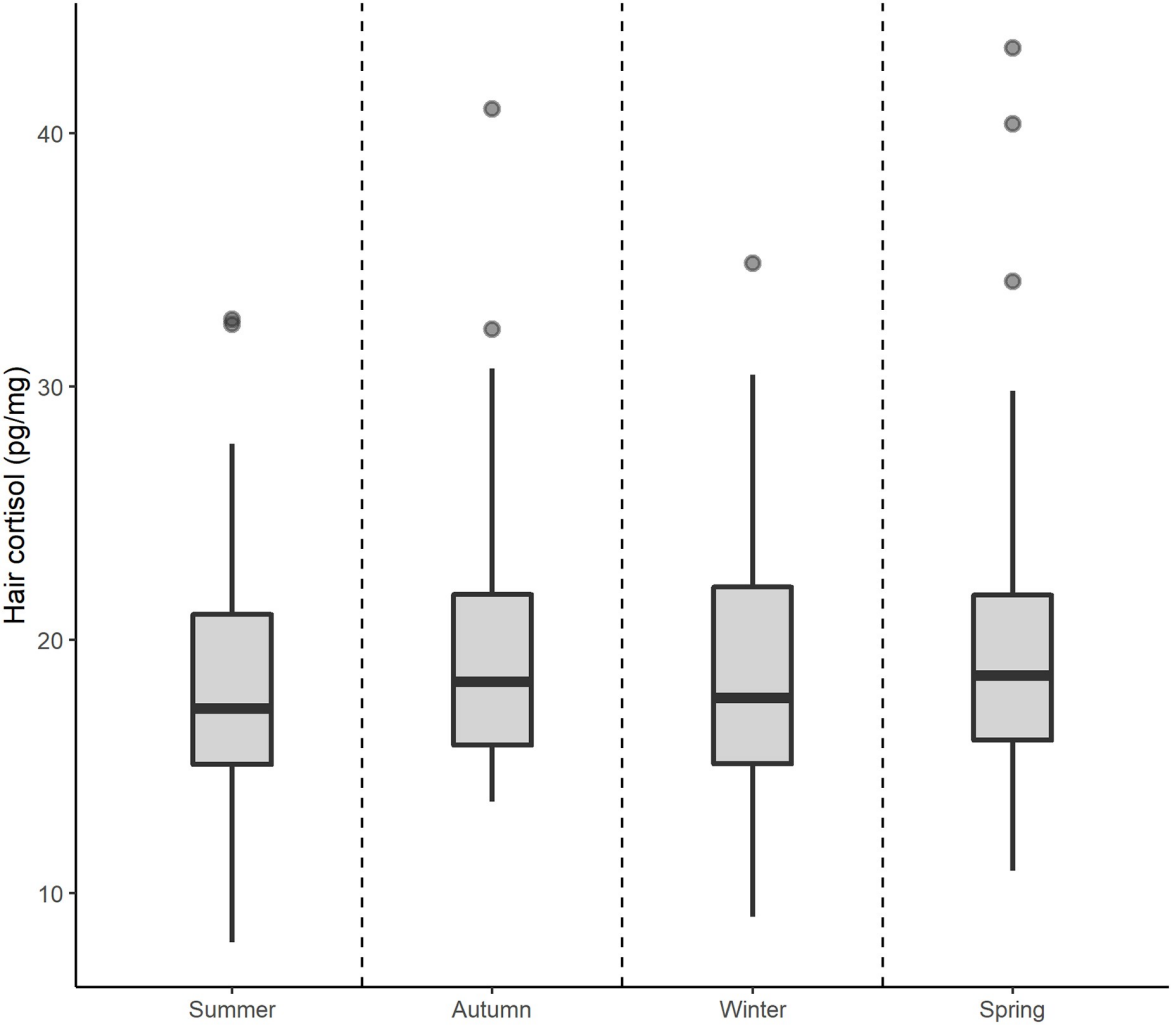
Variation in hair cortisol (pg/mg) in Egyptian mongoose collected in different seasons. Hair cortisol concentration was lower in samples taken in summer than other seasons, although this effect was not significant.

There was no evidence for an interaction between sex and either season or age (Table 1), therefore all further results consider a model without interaction terms included. Hair cortisol concentration varied significantly with mongoose age, with early stage juveniles showing higher hair cortisol concentration than all other age cohorts (Fig 2a; Table 1). Hair cortisol concentration also differed significantly between sexes, with males showing higher hair cortisol concentration than females (Fig 2b; Table 1). There was also a significant change in hair cortisol with storage time, with lower hair cortisol concentration the longer the sample was stored (Fig 2c; Table 1). Hair cortisol concentration did not vary significantly between seasons, although mean hair cortisol concentration from summer samples tended to be lower than those in other seasons (Fig 3; Table 1). Parameter estimates were qualitatively similar between models fitted both with and without the two major outliers (Table 2).

**Table 1:** Significance of model terms calculated using parametric bootstrapped likelihood ratio tests.

Variable	Likelihood ratio test value	p-value
Interaction*	6.40	0.428
**Storage**	**9.51**	**0.020**
**Sex**	**5.05**	**0.028**
Season	5.71	0.187
**Age**	**19.38**	**0.001**
Reproductive state**	2.22	0.362

Likelihood ratio test value is the value of the likelihood ratio test value generated from the true data, which was then compared to likelihood ratio test values simulated with parametric bootstrapping.

*Note that the significance of interactions was determined by comparing a model with two interactions (sex and age, sex and season) to one with no interactions included.

**Significance of all terms except reproductive state are calculated using a model with data from both male and female mongoose. Significance of reproductive state was calculated using a model with data from females only (see section 2.5.2). Significant terms (α = 0.05) are in bold

**Table 2:** Effects of age, sex, season and storage time on hair cortisol concentration of Egyptian mongoose in two models fitted with and without two major outliers.

Variable	Parameter estimate (outliers removed)	[95% confidence interval]	Parameter estimate (outliers included)	[95% confidence interval]
**Intercept**	**33.96**	**[23.35/44.73]**	**32.05**	**[18.12/46.08]**
**Juvenile (I)**	**4.31**	**[2.22/6.41]**	**4.51**	**[0.73/8.22]**
Juvenile (II)	-0.50	[-2.46/1.46]	-0.60	[-4.11/2.85]
Sub-adult	-0.42	[-2.46/1.55]	1.29	[-2.29/4.9]
Autumn	1.63	[-0.83/4.1]	1.56	[-2.93/6.02]
Winter	2.29	[-0.63/5.19]	4.87	[-0.2/9.85]
**Spring**	**2.61**	**[0.26/4.99]**	3.01	[-1.41/7.41]
**Male**	**1.36**	**[0.1/2.61]**	1.35	[-0.86/3.55]
**Storage time**	**-0.01**	**[-0.02/-3e**^**-3**^**]**	**-0.01**	**[-0.02/-7e**^**-4**^**]**

The table shows parameter estimates of a general linear mixed effects model with 95% confidence intervals (estimated with parametric bootstrapping with 5,000 iterations). All parameter estimates where 95% confidence interval does not include 0 are in bold. Adult females in summer are used as the reference level.

The full model, with outliers removed, gave a marginal R^2^ value of 0.19 (variance explained by fixed effects) and a conditional R^2^ value of 0.39 (variance explained by fixed and random effects). Repeatability of all three random effects was low and could not be distinguished from zero in any case after parametric bootstrapping (Repeatability ± standard error: Province 0.09 ± 0.07; Year 0.09 ± 0.12; Month (within year) 0.06 ± 0.05).

### Effect of female reproduction state

**Fig 4:**
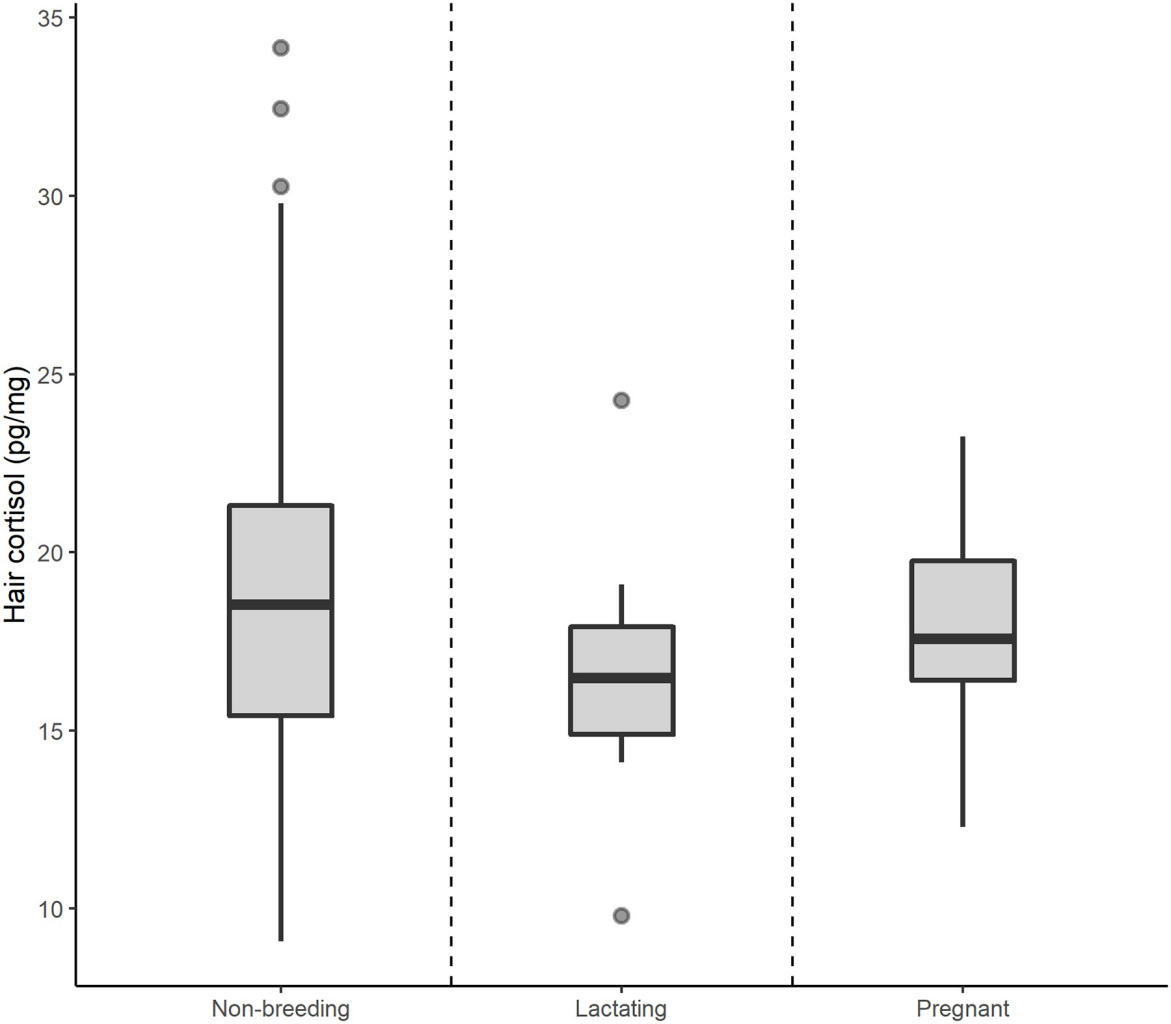
Variation in hair cortisol concentration in female Egyptian mongoose in different reproductive states. Hair cortisol concentrations were similar between reproductive states.

There was no evidence of an effect of reproductive state on hair cortisol in female mongoose (Table 1; Fig 4; Table 3). Effects of age, season and storage time were consistent with analysis conducted with both sexes (Table 3).

**Table 3:** Effects of age, season, storage time and reproductive state on hair cortisol concentration of female Egyptian mongoose.

Variable	Parameter estimate (outliers removed)	[95% confidence interval]
**Intercept**	**31.49**	**[24.14/38.77]**
Lactating	-1.81	[-5.11/1.51]
Pregnant	-1.32	[-3.79/1.27]
**Juvenile (I)**	**2.5763**	**[0.15/5.01]**
Juvenile (II)	-0.6448	[-3.16/1.83]
Sub-adult	-2.1557	[-4.52/0.19]
Autumn	-0.2291	[-2.56/2.05]
Winter	1.6513	[-0.81/4.06]
**Spring**	**2.2434**	**[0.14/4.30]**
**Storage time**	**-0.01**	**[-0.01/-4.6e**^**-3**^**]**

Table shows parameter estimates of a general linear mixed effects model with 95% confidence intervals (estimated with parametric bootstrapping with 5,000 iterations). All parameter estimates where 95% confidence interval does not include 0 are in bold. Lactating adults in summer are used as the reference level.

## Discussion

This study validated an EIA for hair cortisol measurement and characterized its baseline variation in a wild population of Egyptian mongoose. Our analysis encompassed individuals of both sexes and all ages, across a range of geographic, environmental and seasonal conditions that the species experiences in Portugal allowing us to account for spatial, temporal and biological factors that may contribute to hair cortisol variation. Our results showed that age, sex and storage time had an effect on hair cortisol, but season did not. By identifying which factors influence baseline hair cortisol in this wild population, we have established the basis for the application of hair cortisol measurement to understand the effect of natural and anthropogenic stressors.

Following methanol extraction, glucocorticoid metabolites (GCM) in a pooled hair sample were characterized by high-performance liquid chromatography (HPLC) and enzyme immunoassay (EIA). One major peak co-eluting with the cortisol standard was present. Besides cortisone, two unknown immunoreactivities were detected at positions not coinciding with one of our available steroid standards. This agrees with data in guinea pigs from Keckeis et al. [47], who found cortisol, cortisone and corticosterone as well as unknown immunoreactivities, when applying EIAs for cortisol and cortisone. Particularly, our polar immunoreativities in fractions 3–5 were also found by these authors. Unknown cortisol-like immunoreactivities were also found in sheep by the same group [55], with different amounts depending on the selected EIA. Altogether our results revealed our cortisol assay is a suitable diagnostic tool for quantifying cortisol in hairs from the mongoose.

Age significantly influenced hair cortisol concentration in Egyptian mongoose, with early stage juveniles, between two-and-a-half and five-and-a-half months, exhibiting higher levels of hair cortisol than other age cohorts (Fig 2a). This age cohort includes the recently weaned mongoose pups [56] and is consistent with the post-weaning cortisol increase found in the southern elephant seal [33]. Elevated glucocorticoid secretion during infancy has been described using faecal glucocorticoid metabolite analysis in baboons [57] and hair cortisol in non-human primates [58] and humans [59]. However, our present findings are inconsistent with hair cortisol data from other wild carnivores, such as grizzly bears [12], polar bears [60] and the Canada lynx [14], where no effect of age was detected. This difference could be related to differences in hair growth and moult patterns between species during their juvenile development. While diffusing into the hair during active growth, the amount of cortisol incorporated could be influenced by different types of juvenile coats and rapid growth and moult rates, and this effect could be even more pronounced in the presence of increased circulating cortisol levels. Unfortunately, we know very little about hair growth and moult patterns of wild species, including the Egyptian mongoose. An alternative explanation could be that age classification using dental growth allowed a finer scale of age classification in our study by separating age cohorts that are otherwise morphologically similar. On the other hand, bears and lynxes, as apex predators, are unlikely to be subject to predation or aggressive interspecific interactions. In comparison, the Egyptian mongoose, a meso-predator, experiences aggression and killing by sympatric carnivores like the Iberian lynx, wild domestic dogs [56,61,62] or large raptors [63], which could result in elevated cortisol levels. The characterization of hair growth and moult patterns and comparison of developmental patterns of hair cortisol in sympatric apex- and meso-predators would help clarify these questions. Analysis of hair cortisol in Iberian lynx, Egyptian mongoose, and other species of small carnivores in Portugal would provide an ideal system for such work.

Our data also support an effect of sex on hair cortisol concentration, with males showing higher cortisol concentration than females. This effect was not observed in previous studies in reindeer [9], grizzly bears [12] or Canada lynxes [14]. Investment in reproduction differs substantially between sexes in Egyptian mongoose and annual peaks in body condition and spleen weight are different between sexes and coincide with moments of investment in reproduction [45,46]. Egyptian mongoose males need to maintain and defend large territories [43] with frequent aggressive encounters with conspecifics [64] which might explain the observed sex differences in hair cortisol. On the other hand, the effect of sex could also be explained by the attenuation in HPA-axis reactivity in breeding females in order to avoid diverting resources from reproduction, as described in other species [65]. Either way, we would expect these effects to be seasonal, and therefore visible in the interaction of sex with season of our model; however, this was not the case. Indeed, there was no significant effect of season or the interaction of season with sex, which questions these explanations. It is possible that the effect is simply due to differences in hypothalamic-pituitary-adrenal axis function between sexes [32,66–68] in this species.

Season had no effect on hair cortisol levels in our study. However, a knowledge gap on hair cycles in wild mammals limits our ability to determine to which periods our hair cortisol values refer to. Wild terrestrial mammals in arctic and temperate climates usually undergo two yearly moults, one in spring and one in autumn, that do not overlap with periods of reproductive activity [69,70]. The retention of the previous pelage throughout reproduction could be the reason no changes in hair cortisol levels were found in our data during the reproductive period of the Egyptian mongoose. Hair cycles have been described previously in humans, domestic and laboratory species, pinnipeds and mustelids, but the variability in hair cycles even between closely related species [69] impedes extrapolation to the case of the Egyptian mongoose. Early observations do not support the existence of seasonal differences in coat length in this species in Israel [56]. Assuming this species moults at least once a year, we would expect to find an effect on hair cortisol due to changes in incorporation rate in the seasons when moults occurred, but we did not. This raises important questions regarding the interpretation of hair cortisol levels in wild mammals in general, and prompts research focusing on the effect of hair cycles in order to allow us to fully interpret our results on a temporal scale. While a confounder at this point, this information is a potential strength in the application of hair cortisol as a marker of stress once the effect of hair cycles is understood, because the signal of elevated cortisol levels during major life events might remain detectable for several months.

Storage time had a negative effect on the amount of cortisol retrieved from hair. Previous studies in animals have seen no influence of storage time on hair cortisol when intact hair is stored at room temperature for over one year [11,12], and based on a study in Peruvian mummies [71] it is presumed to remain detectable for longer periods. However, an effect of storage on grizzly bear hair stored in ground form was observed over a period as short as nine months [12], suggesting that hair integrity plays an important role. The longer duration of storage and the effect of freezing on hair integrity are possible explanations for the presence of this effect in our study. Our samples were stored between 863 to 2,266 days before processing, which four-fold exceeds the storage time assessed in grizzly bears [12] and dairy cattle [11]. Since the decrease in hair cortisol over time should occur very slowly, this small effect is more likely to be detected in studies focusing on longer periods and where a broad range of factors with potentially larger effects such as sex, age, geographic and temporal variation, are accounted for. Finally, part of the storage time of our samples was spent in the form of frozen cadavers before thawing and collection of hair samples, which could also have caused loss in hair integrity. The other possibility is the existence of a true variation in hair cortisol concentration of these animals due to changes in biotic or abiotc factors (e.g. predator-prey cycles) over the four years of the sampling period. This explanation was controlled for by the inclusion of month and year as random factors in model construction.

We expected lactating and pregnant females to exhibit higher baseline adrenocortical activity, and consequently more cortisol in hair. However, reproductive state was not significant in our female-only model. This could be explained by the small number of reproductively active females in our sample, to the difficulty in accurately detecting lactation or early pregnancies during dissection of previously frozen specimens, or simply by the fact that some females may have similarly high levels of hair cortisol due to factors we have not accounted for in our model. Despite non-significant, looking at the raw data (Fig 3), lactating and pregnant females tend to have lower hair cortisol concentration when compared to non-breeding females. According to studies in different mammal species, baseline HPA-axis activity is decreased during the initial period of pregnancy but steadily increases during the second and last thirds of pregnancy, peaking at parturition [65,72]. Meanwhile, HPA-axis reactivity to stress is supressed throughout the entire pregnancy [73]. A similar trend is seen during lactation, where an increase in baseline activity is accompanied by attenuated HPA-axis reactivity [65,74]. Because hair incorporates blood cortisol from baseline levels and stress-induced responses altogether, our results suggest that, in breeding females, the net effect of the increase in baseline and supressed reactivity resulted in total cortisol levels similar to those of non-breeding females.

## Conclusion

We investigated the variation of hair cortisol in a wild population of Egyptian mongoose in Portugal. Our results describe the baseline variation in hair cortisol in this population and highlight the importance of accounting for influences of age, sex and storage time when using hair cortisol. With this information, future studies should be able to apply hair cortisol measurements as a non-invasive technique to study effects of natural and anthropogenic stressors in wild mammals.
