# Correction: Age, sex and storage time influence hair cortisol levels in a wild mammal population

**DOI:** 10.1371/journal.pone.0222963

**Published:** 2019-09-18

**Authors:** Alexandre Azevedo, Liam Bailey, Victor Bandeira, Martin Dehnhard, Carlos Fonseca, Liliana de Sousa, Katarina Jewgenow

The affiliation for the second author is incorrect; the correct affiliation is not indicated. Liam Bailey is not affiliated with #2, but with: Department of Evolutionary Genetics, Leibniz Institute for Zoo and Wildlife Research, Berlin, Germany.
